# 3*d* Transition Metal Adsorption Induced the valley-polarized Anomalous Hall Effect in Germanene

**DOI:** 10.1038/srep27830

**Published:** 2016-06-17

**Authors:** P. Zhou, L. Z. Sun

**Affiliations:** 1 Hunan Provincial Key laboratory of Thin Film Materials and Devices, School of Material Sciences and Engineering, Xiangtan University, Xiangtan 411105, China; 2 Key Laboratory of Low-dimensional Materials and Application Technology, School of Material Sciences and Engineering, Xiangtan University, Xiangtan 411105, China

## Abstract

Based on DFT + U and Berry curvature calculations, we study the electronic structures and topological properties of 3*d* transition metal (TM) atom (from Ti to Co) adsorbed germanene (TM-germanene). We find that valley-polarized anomalous Hall effect (VAHE) can be realized in germanene by adsorbing Cr, Mn, or Co atoms on its surface. A finite valley Hall voltage can be easily detected in their nanoribbon, which is important for valleytronics devices. Moreover, different valley-polarized current and even reversible valley Hall voltage can be archived by shifting the Fermi energy of the systems. Such versatile features of the systems show potential in next generation electronics devices.

Besides charge and spin degree of freedoms, valley degree of freedoms of electrons has attracted intense interest recently due to its potential in next generation electronics devices, now called as valleytronics^1^,^2^,^3^,^4^,^5^. The valley represents a local maximum/minimum on the valence/conduction band of certain semiconductors inside the first Brillouin zone. The different valley can be regarded as a discrete degree of freedom for low-energy carriers, which is robust against smooth deformation and low-energy phonon thanks to the large separation of the valleys in momentum space^3^. Recently, much theoretical and experimental works reported the valleytronics in a variety of systems, including three dimensional diamond^6^ and Bi^7^, two dimensional single layer Transition metal dichalcogenide^3^,^8^, graphene^2^,^9^,^10^ and silicene^4^, one dimensional carbon nanotube^11^ and zero dimensional Si quantum dot^12^. Among these optional materials, two dimensional (2D) honeycomb lattice materials is one of the most promising candidates for future increasing miniaturized electronic device. Because its valley can be easily controlled by external conditions and the valley K and *K*′ can be encoded traditional 0 and 1^10^,^13^. Recently experiment proved that the dependence of the anomalous Hall conductivity on photon helicity can be archived in 2D *MoS*_2_^8^. It pave the way for futures device application of valleytronics. To further utilize the valley index as an information for carrier, generation, detection, and identification of pure valley current and their response to external stimuli are therefore crucial premise.

Graphene provides an excellent platform to make use of this freedom^2^. Valley-polarized phases can be acquired by breaking its space inversion symmetry. Although massive theoretical works have proposed for the valley-polarized phenomenon in graphene, it is hard to realize in experiments due to its flat structure and weak spin-orbit coupling of C atom. This predicament has motivated researchers to investigate two-dimensionally ordered and layered materials analogous to graphene, such as silicene or germanene. Silicene and germanene have recently attracted much attention both in theoretical and experimental fields^4^,^14^,^15^,^16^,^17^,^18^,^19^ due to their versatile properties. Different from flat graphene, both silicene and germanene share buckled hexagonal lattice. It is easier to destroy their inversion symmetry and produce valley polarized phenomenon in the materials. The pioneering work of Ezawa^4^ have proved that many special valley-polarized topological phase would appear when proper external electric field and exchange field applied to silicene. An recent review about germanene^20^ also indicated that it is potential candidate for future 2D nanoelectronics. Using first-principles method, Cahangirov *et al.*^21^ show that germanium atoms can form two-dimensional low-buckled honeycomb structures, germanene. They also indicate that charge carriers of germanene behave as a massless Dirac fermion. Subsequent study^22^ indicated that quantum spin Hall effect can be realized in germanene. Latest theoretical research also reported that functionalized germanene behaves as large-gap two-dimensional topological insulators^23^. In experiments, Bianco *et al.*^24^ indicated that millimeter-scale crystals of hydrogen-terminated multilayered germanene can be obtained from chemical deintercalation of *CaGe*_2_, and single layer germanene can be obtained from exfoliating the material. However, its semiconductor properties with nearly 1.59 eV bandgap at Γ point hinder the utilization of its Dirac electron. Pristine germanene on the surface of Pt (111), Au (111), and Al (111)^14^,^15^,^25^ was recently synthesized. They were reported to be 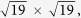

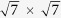
 or 3 × 3 reconstruction on the substrates. However, it is worthy to point out that on Pt (111) surface the germanene maybe a kind of surface alloy composed of *Ge*_3_*Pt* tetramers^26^. An recent experiment also reported that germanene terminated *Ge*_2_*Pt* clusters can be grown on the Ge (110) substrate^27^. So it is suggested that detail electronic structure detection should be done to these synthesized germanene. It is urge to determinate whether the electronic properties of these germanene are similar with free-standing one.

The germanene produced on the aforementioned metal substrates shows weak buckled structure, and no flat gernamene was observed. Although the weak buckled configuration observed in the experiments is little different from the lower buckled model used in present work, theoretical work^21^ indicated that by comparing with planar honeycomb structure, lower buckled germanene is dynamic stable. Moreover, the most prospective property for germanene is its Dirac electrons and the metal states of the substrates would absolutely cover up this exciting property. Therefore, to use this fascinating properties of germanene, it should be grown or transferred to more inert and insulator substrate, such as 2D BN or AlN 20,28,29, or form the van der Waals (vdW) heterostructures^28^,^29^ with these 2D wide band gap materials. Considering such substrates will largely reduce the impact on the structure of germanene, we expect it will show the buckled structure as discussed in our present work.

Lately, valley-polarized quantum anomalous Hall effect (QAHE) was reported in silicene within tight-binding frame^30^. Anomalous Hall effect (AHE) is represented by anomalous Hall conductance occurring in magnetic materials^31^. Latest calculations also predict quantum AHE can be realized in Co-decorated silicene^18^. The valley-polarized quantum anomalous Hall insulator possesses quantum valley Hall effect (QVHE) and QAHE at the same time. Namely, it possesses the non-dissipative anomalous hall transportation and an additional new valley freedom. This new phase broadens the application of valleytronics in next-generation electronic devices.

Inspired by the advancement of both germanene and QVHE, in this work, we concentrate on how to adjust the valley-polarization of germanene by introducing 3*d* transition metal (TM) atom on its surface. In comparison with previous reports, we find that appropriate Hubbard U is important to accurately describe the TM-germanene systems. With the help of first-principles Berry curvature calculations, we find that 3*d* TM atom can break the inversion symmetry of germanene and induce valley-polarization when the spin-orbit coupling (SOC) is included. Valley-polarized AHE can be achieved when Cr, Mn, or Co is adsorbed on the surface of germanene. Moreover, quantum valley Hall effect can be realized in Mn-germanene system just through shifting the Fermi level to specific energy window.

## Results and Discussions

**Figure 1:**
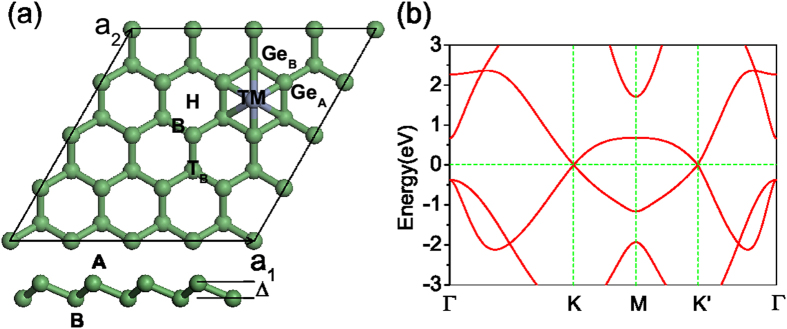
(**a**) Top view of germanene monolayer where 3 adsorption sites (Hollow (H), top B sublattice (*T*_*B*_) and Bridge (B)) are marked out with black letters. The lower panel of (**a**) is the side view of germanene, the two equivalent Ge sublattices are labeled as A and B, respectively, with a buckled distance Δ. (**b**) The energy band of pristine germanene. Two equivalent valleys at *K* and *K*′ clearly locate around the Fermi level.

Germanene consists of a honeycomb lattice of germane atoms with two sublattices A and B, as showed in Fig. 1(a). Different from the strong *π* bond of graphene, the weak *π* bond of germanene, which mainly comes from *p*_*z*_ states of Ge and more delocalized than that of C, is not enough to stabilize planar structure. The germanene shows a kind of buckling structure with A/B sublattices. Based on previous both experimental and theoretical reports^14^,^15^,^21^, we considered the lower buckled germanene in present work. Similar to graphene, its lower energy electronic structure around Fermi level is mainly dominated by two equivalent Dirac cone at *K* and *K*′, namely two valleys as shown in Fig. 1(b). This equivalency is protected by the inversion symmetry of the A and B sublattices. If this symmetry is broken, valley-polarization will appear. Before investigating the system of TM adsorbed germanene, we perform the optimization calculations of the pristine germanene. The crystal constant and buckling distance are respectively 4.02 Å and 0.61 Å, which is in good agreement with previous reports^21^,^22^.

Then we study the system of TM adsorption on the germanene. In present work, we consider Ti, V, Cr, Mn, Fe, and Co as the TM adsorbates. Since the adsorption of Ni atom does not induce spin-polarization in germanene (time reversal symmetry broken induced by spin-polarization is a prerequisite for the intrinsic AHE), moreover, the valley-polarization of Ni-germanene is not obvious, considering the essential topic in this paper, we do not consider Ni-germanene in present work. Previous report by Wehling *et al.*^32^ indicated that electronic structures, adsorption geometry, and magnetic state of the 3*d*-TM adsorbed graphene are very sensitive to the treatment of the local Coulomb interactions U of the TM *d* orbital. Moreover, the first-principles Hubbard U is important to accurately describe the electronic structure of 3*d* TM absorbed germanene due to the correlation of 3*d* electrons is strongly dependent on its occupation and surrounding. Using the linear response approach^33^ we calculate the Hubbard U of all the six TM atoms adsorbed on germanene (the details are provided in supplementary materials), the values of U are listed in Table 1. The calculated results of the six kinds of 3*d* TM adsorbed germanenes based on the Hubbard U obtained above are also listed in the Table 1. To obtain the favorable site of single TM atom adsorption on the surface of germanene, we calculate the adsorption energy on three adsorption sites under spin ground state for each TM, including hollow, bridge, and topB of germanene as shown in Fig. 1(a), the formula of adsorption energy is stated in the method section. In general, the total energy and adsorption energy of TM and TM-germanene will depend on the parameter U. Therefore, it is not meaningful to compare the adsorption energies resulting from the calculations with different U^38^. In order to compare the stable trend of different TM atom, we firstly use GGA method without U. The adsorption energy indicates that the hollow absorption site is the most stable configuration for all the six TM-germanene systems. It is worthy to mention that the favorable site for single TM adsorption does not depend on the the Hubbard U. However, as pointed out by previous works^34^,^35^, the absolute adsorption energy depends on the spin state of TM which will be influenced by the Hubbard U. Therefore, we also calculated the adsorption energy with the parameter U obtained by the linear response theory. The adsorption energies with and without Hubbard U are listed in Table 1. The results indicate that the adsorption energies either with or without U are all higher than 1.0 eV indicating chemical adsorption of the TM. However, the adsorption energy with U generally higher than that without U except for Cr. Such results on the one hand derives from the localization effect of the d orbital produced by the U, which will weaken the bonding effect between the d orbital of TM and p orbital of Ge. On the other hand, the U will influence the spin state, which will change the bonding nature as reported in previous works^34^,^35^. From the comparison of total magnetic moment to TM-germanene with and without Hubbard U, we find that, the total spin moment of composite system increase except Cr. The magnetic moment with and without Hubbard U can be found in supplementary materials. After comparing the PDOS for TM-germanene systems between without (Fig. S6) and with Hubbard U, we found such magnetic moment increase derives from the increasing filling of their spin-down electronic states around Fermi level and, shifting the states to higher energy will produce the increase in the adsorption energy. Beside the change of magnetic moment, another obvious influence of Hubbard U is narrowing the energy width of occupied spin-up states. However, for Cr-germanene, its PDOS as shown in Fig. S6 shows that the effect of Hubbard U narrows the energy band width of *E*_1_ and *E*_2_ as well as shifts *A*_1_ above the Fermi level. The two effects finally make the Cr change from the high spin state (4.47 *μ*_*B*_) to low spin states (2.84 *μ*_*B*_). Consequently, its adsorption energy decreases abnormally with Hubbard U. The absorption energies for all TM-germanene systems without Hubbard U range from 1.489 eV to 4.807 eV which is much larger than that of TM-graphene^36^ (less than 1.0 eV). The results indicate that 3*d* TM atom adsorbed germanene shows strong stability. For V, Cr, and Fe, the absorption energy agrees well with the results of uniform Hubbard U = 4 eV^37^, and the difference is only around 0.1 eV. However, the adsorption energy of Ti, Mn and Co is 0.35, 1.43, and 0.93 eV larger than that of uniform Hubbard U = 4 eV^37^, respectively. This difference mainly attributes to relatively larger Hubbard U in our calculations. Although the comparison of the adsorption energy between different Hubbard U is not that much meaningful, our recent work^38^ indicates that appropriate on-site Hubbard U can even influence the spin-polarized ground state. The calculated magnetic moments based on the Hubbard U derived from the linear response approach^33^ show great difference from previous report with uniform Hubbard U^37^. The difference derives from the different electron occupation in the *d* orbital which is sensitive to the on-site Hubbard U. For instance, when the Hubbard U = 4.666 eV the electrons of Ti-germanene almost averagely occupy the majority spin of 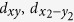 and 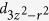. Therefore the total magnetic moment is close to 3 *μ*_*B*_. However, when we take the Hubbard U = 4.0 eV the valence electrons fill only in the majority spin of *d*_*xy*_ and *d*_*xz*_ resulting in the magnetic moment close to 2 *μ*_*B*_.

To check the clustering effect of 3d transition atoms on the surface of germanene, we take the Cr-germanene as an example to obtain the adsorption energy of another extra Cr atom adsorbs close to Cr of Cr-germanene system. Here we choose four initial top position. The details about the configuration and computational details can be found in supplementary materials. The results show that although the dimer of TM atom can be form on the surface of germanene, we expect the clustering formation would not damage the valley-polarized AHE. However, the concrete AHE would change with the formation of TM cluster. All in all, although the TM dimer can form, the valley-polarized AHE modulation to germanene still exists. In our present work we only consider the TM dimer on the effect of the valley-polarized AHE in germanene. The conclusion of TM dimer will be suitable for the low concentration of the TM adsorption. According to high concentration of TM adsorption, large TM cluster on the valley-polarized AHE of germanene is worth studying, however, considering the complicated of large TM cluster configuration on the surface of germanene, it does not include in present work.

As mentioned above, pristine germanene consists of A and B sublattices with different height in the buckled configuration. The foreign TM adatom shows different distance with the nearest neighbor (NN) two sublattices d (*TM*–*Ge*_*A*_) and d (*TM*–*Ge*_*b*_), as listed in Table 1. The difference of these two distances is around 0.2–0.5 Å. Thus, a local staggered AB-sublattice potential will be induced by the TM adatom. This effect is similar to vertical electric field applied to a single layer silicene^39^,^40^. The two valley *K* and *K*′ become un-dependent after the TM adsorption. The charge transfer *T*_*e*_ between TM and germanene as shown in Table 1 indicates that all TM-germanenes are n-type doped by the TM atom. The charge transfer decreases from Ti (1.153e) to Co (0.285e) attributing to the increase in the electronegativity from Ti to Co. The n-type doping of TM on germanene will be further discussed below.

**Figure 2:**
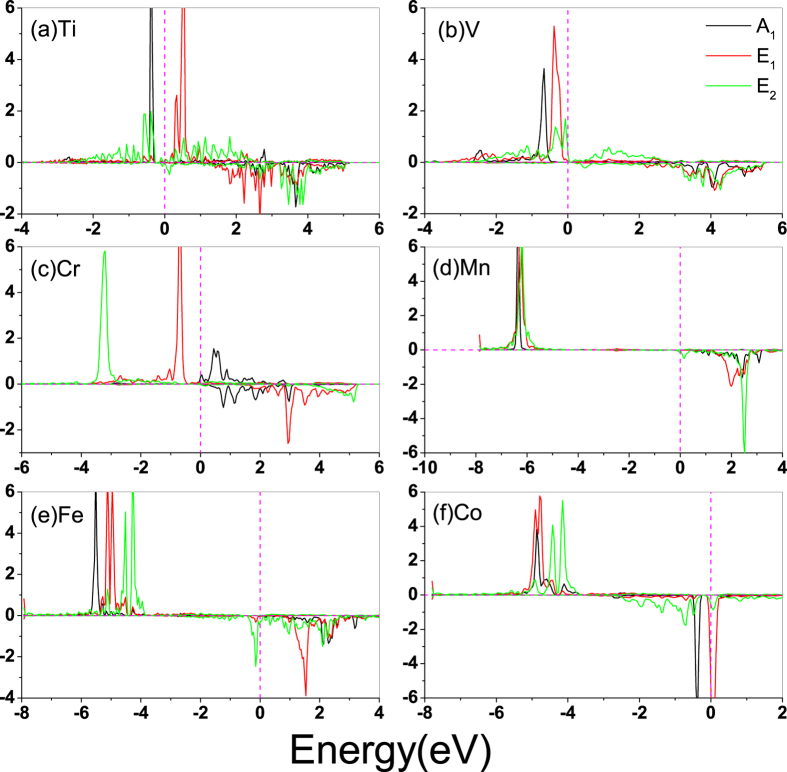
Projected density of states for TM-germanene system with Hubbard U. The positive and negative values denote spin-up and spin-down channels, respectively. The Fermi energy is set to zero.

**Figure 3:**
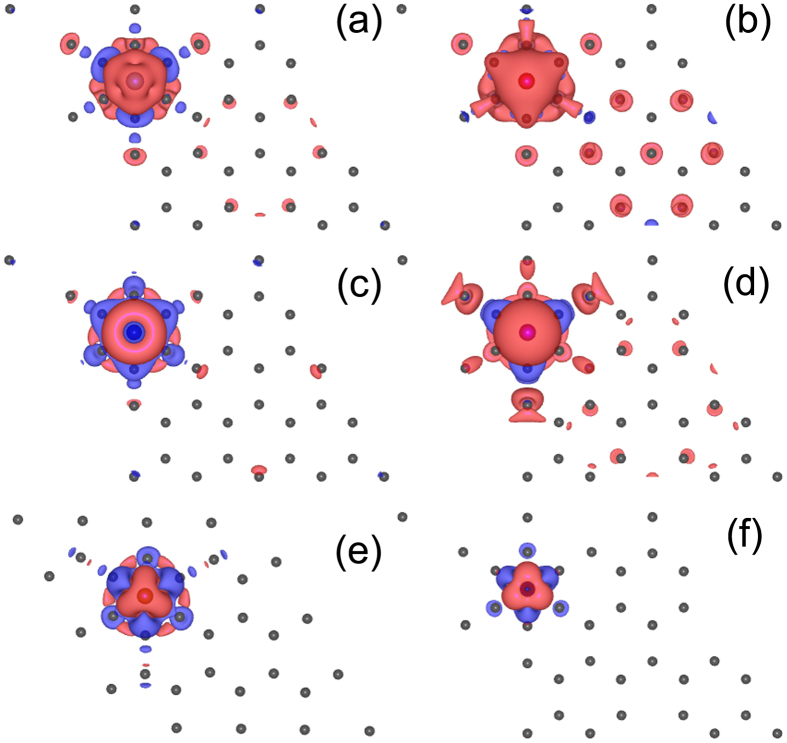
Spin charge density distribution of (**a**) Ti-germanene, (**b**)V-germanene, (**c**) Cr-germanene, (**d**) Mn-germanene, (**e**) Fe-germanene, and (**f**) Co-germanene.

To understand the magnetic properties of TM-germanene as listed in Table 1, we plot the partial density of states (PDOS) of TM-germanene systems without SOC in Fig. 2 and spin charge density (SCD, defined as the difference between spin-up and spin-down charge density) as shown in Fig. 3. When the TM atom adsorbed on the hollow site, the inversion symmetry in point group *D*_3*d*_ of the pristine germanene is broken and the symmetry around the hollow site is reduced to *C*_3*v*_ through lengthen the distance between its three NN Ge atoms belonging to same sublattice. As shown in Fig. 1, although the TM lengthen the distance of the nearest neighbor Ge-Ge bond to TM atom, the 120 degree rotation operation is still remained for both sublattices. The symmetry around the TM adsorbed hollow site shows *C*_3*v*_ characteristics. Therefore, we can qualitatively split the 3*d* sub-shell of adatom into three groups: *A*_1_ symmetry group only included 3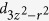 state; the twofold degenerate *E*_1_ group consisted of 3*d*_*xz*_ and 3*d*_*yz*_; 3*d*_*xy*_ and 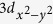 made up of the *E*_2_ group. Such analysis is equal to the consideration of each *d* orbital. However, as showed in Fig. 2 the occupied *d* groups and spin-polarization almost share the same feature, namely the SCD comes from all *A*_1_, *E*_1_ and *E*_2_ group except for Cr-germanene only comes from *E*_1_ and *E*_2_ group. So it is hardly to distinguish which group contributes to the SCD. Then we only concentrate on the magnetic interaction between total *d* orbitals of TM and its NN Ge atom. For the case of Ti-germanene, the strong spin splitting almost makes the minority states totally locate in the conduction band, and the occupied double degenerate majority *E*_2_ and majority *A*_1_ give rise to 2.67 *μ*_*B*_ magnetic moment in the system which is nearly two times larger than that of uniform Hubbard U^37^. The spin charge density of Ti-germanene, as shown in Fig. 3(a), indicates that the Ti shows anti-ferromagnetic coupling with its NN A site Ge and ferromagnetic coupling with its NN B site Ge. It is worthy to mention that, differing from the extended distribution *E*_1_ and *E*_2_, the dumbbell-shaped *A*_1_ is localized, indicating less interaction with neighboring Ge atoms. For V-germanene, the PDOS shares similar characteristics with those of Ti-germanene. The major difference is that the majority *E*_1_ of V shows distribution below the Fermi level. The magnetic moment of V-germanene is close to 5.0 *μ*_*B*_ (4.67*μ*_*B*_). The SCD as shown in Fig. 3(b) indicates that the V shows ferromagnetic coupling with both its NN A and B site Ge. For Cr-germanene, some sizable Ge 4*s*4*p*-minority states distribute in the energy window of (−2.0 eV, −3.0 eV) implying a kind of antiferromagnetic coupling between Cr and Ge atoms, which lowers the total magnetic moment of Cr-germanene to 2.84 *μ*_*B*_ in the unit cell. The magnetic moment is smaller than previous report (4.0 *μ*_*B*_)^37^. The SCD as shown in Fig. 3(c) indicates that the Cr shows anti-ferromagnetic coupling with both its NN A and B site Ge. The magnetic moment of Mn-germanene shows amazingly 5.75 *μ*_*B*_. After carefully examined the total DOS and partial DOS, we find that majority *E*_1_, *E*_2_, and *A*_1_ states of Mn are fully occupied below the Fermi level resulting in the magnetic moment of the Mn close to 5.00 *μ*_*B*_. The fully occupied *A*_1_ state is clearly shown in the Fig. 3(d), which is perpendicular to the germanene plane with dumbbell-shape. The SCD as shown in the Fig. 3(d) indicates that Mn shows anti-ferromagnetic coupling with its NN A site Ge and ferromagnetic coupling with its NN B site Ge. Moreover, the next nearest neighbor (NNN) A and B site Ge atoms also show spin polarization same to the Mn adatom producing 5.75 *μ*_*B*_ of the system. In the case of Fe-germanene, the magnetic moment of Fe adatom is close to 3.9 *μ*_*B*_ because its majority states are fully occupied and only small fraction of minority *E*_2_ and *E*_1_ states are occupied just below the Fermi level, as shown in Fig. 2(e). However, the SCD as shown in Fig. 3(e) indicates that the Fe is anti-ferromagnetic coupling with both its NN A and B site Ge, reducing the magnetic moment of the Fe-germanene to 3.04 *μ*_*B*_. Moreover, the dumbbell-shape *A*_1_ state is totally destroyed due to the strong coupling between *A*_1_ state of Fe and *p*_*z*_ state of Ge. For the last case of Co-germanene, the majority states of TM are fully occupied. But minority states *A*_1_ and *E*_2_ are also partially occupied locating below the Fermi level. The SCD as shown in Fig. 3(f) indicates that the Co is anti-ferromagnetic coupling with both its NN A and B site Ge. Moreover the dumbbell-shape *A*_1_ state of Co is partly remained. The total magnetic moment of Co-germanene is 2.03 *μ*_*B*_. It is worthy to mention that because of the 4*s* states of 3*d* TM is extended, the electron of 4*s* easily transfers to 3*d* orbits or 3*s*3*p* orbitals of Ge atoms. Such electron transfer also affect the magnetic performance of TM adatom on germanene.

**Figure 4:**
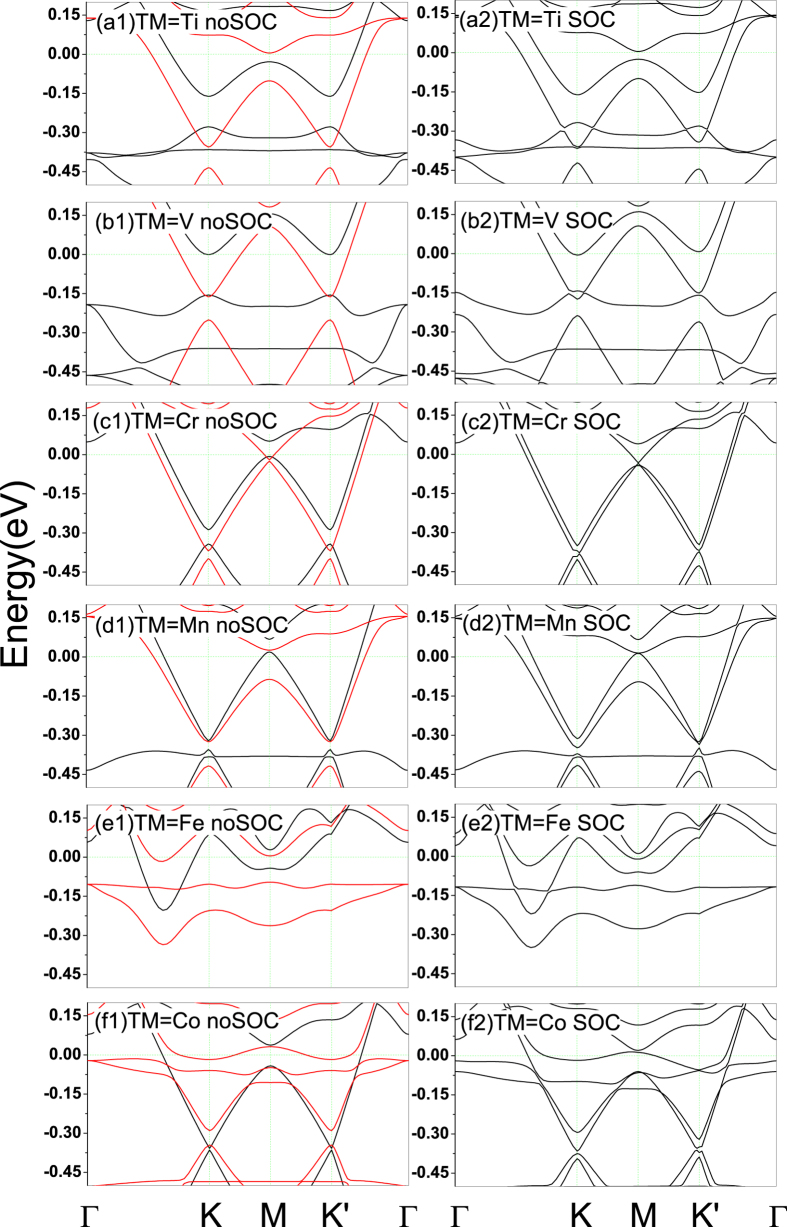
Left column, (a1–f1): the calculated band structures for TM-germanene without SOC. The red and black curves represent the spin-up and spin-down channels, respectively. Right column, (a2–f2): the calculated band structures for TM-germanene with SOC.

Now we concentrate on the energy band structures of TM-germanene without/with SOC. As is showed in Fig. 4(a1–f1), we find that the Dirac cones approximately remain except for Fe-germanene. Since the paper focuses on the valley-polarized phenomenon, Fe-germanene is excluded in later discussions. The Dirac cones of all TM-germanenes shift below the Fermi level due to the electron transfer from TM atom to germanene. Spin-splitting appears around the Dirac cone after the TM atom adsorbed on the hollow site of germanene, which is the prerequisites of AHE around the Dirac cone. The energy band structures with SOC are showed in Fig. 4(a2–f2). Valley-polarized phenomenon is very evident for all TM-germanene systems due to the energy spectrum around Dirac cone exhibiting different characteristics between valley *K* and *K*′. For Ti-germanene and V-germanene, some states other than *K* and *K*′ appear around Dirac cones and cross with the two valleys. Together with the results of PDOS, we confirm that they mainly come from *d* orbits of TM. These localized states will ruin the valley-polarized AHE of Ti/V-germanene, which is proved by Berry curvature calculations below. In comparison with other TM-germanene, we find that the Dirac cones of Cr-germanene and Co-germanene remain very well. After considered SOC, the *K* of Cr-germanene and the *K*′ of Co-germanene show energy band cross feature. For Mn-germanene, a local state derived from the *d* states of Mn crosses through both valleys. Interestingly, there is a global gap around the two Dirac cones for Mn-germanene. The global energy gap is hoping to be the prerequisite for the system showing quantum valley Hall effect, the details will be discussed below.

**Figure 5:**
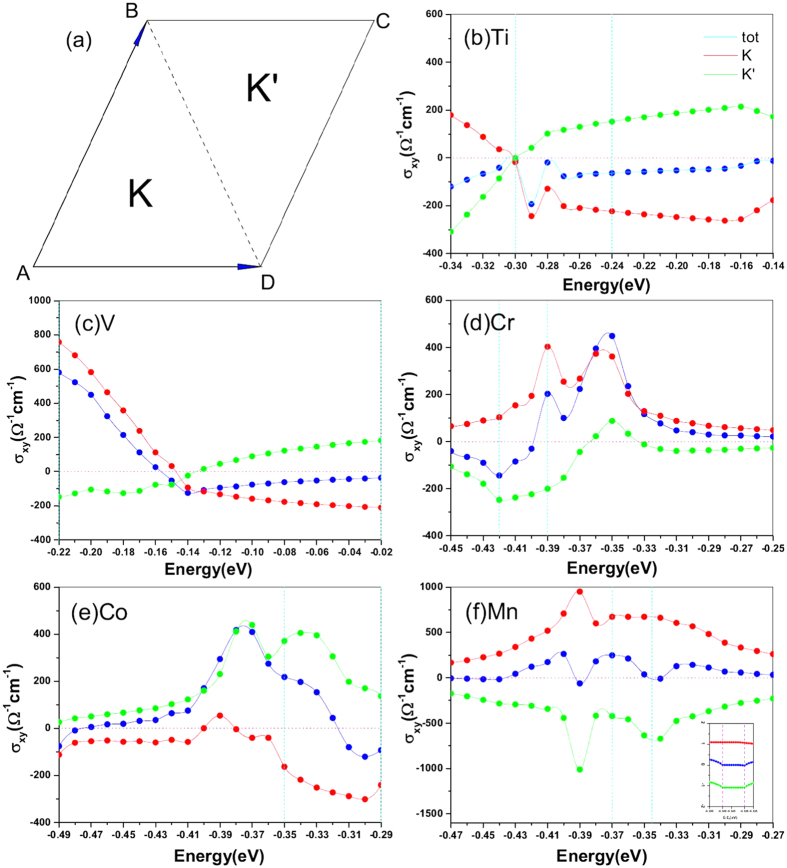
(**a**) The first Brillouin zone for 4 × 4 germanene. AHC around the Dirac cone for (**b**) Ti-germanene, (**c**) V-germanene, (**d**) Cr-germanene, (**e**) Mn-germanene, and (**f**) Co-germanene. The Fermi energy is set to zero. The vertical dotted line represents the energy that we plot the Berry curvature distribution in Fig. 6. The inset of (**f**) represents close inspect the AHC for Mn-germanene near the energy of −0.345.

**Figure 6:**
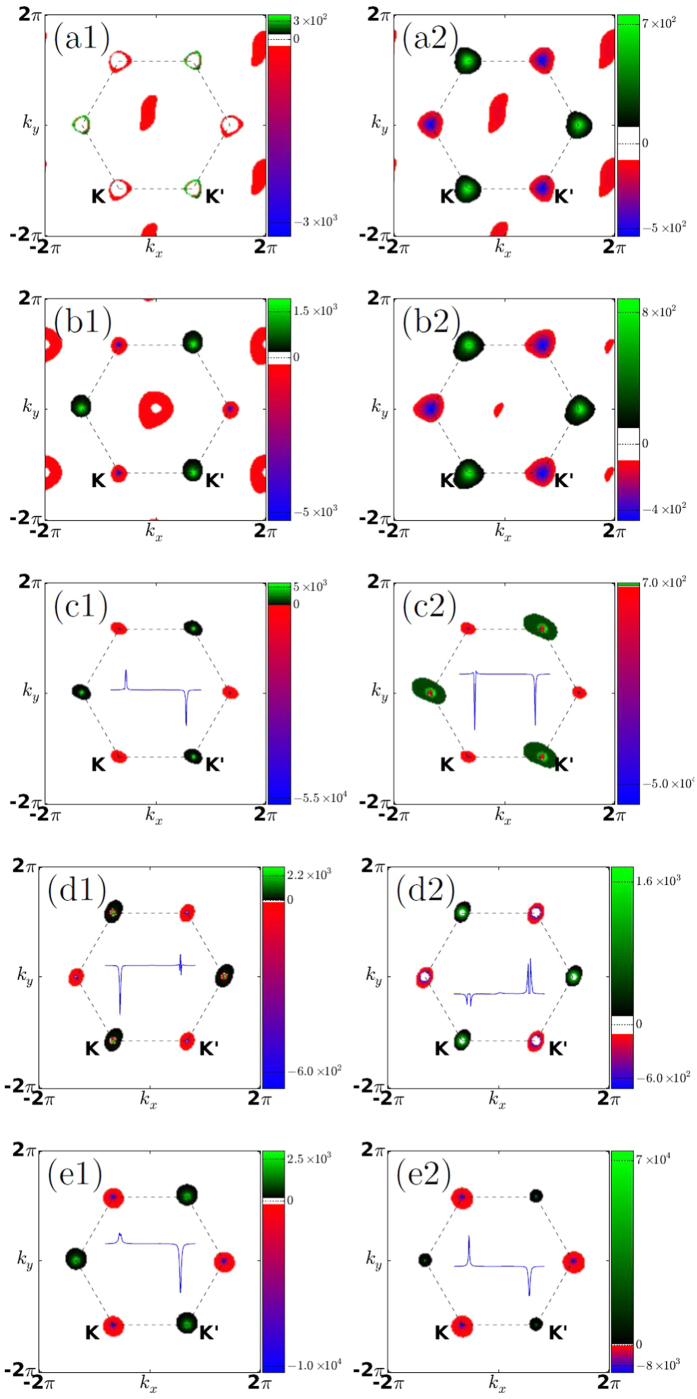
The distribution of the Berry curvature in momentum space for Ti-germanene in the energy of (a1) −0.3 and (a2) −0.24, V-germanene in the energy of (b1) −0.22 and (b2) −0.02, Cr-germanene in the energy of (c1) −0.39 and (c2) −0.35, Co-germanene in the energy of (d1) −0.35 and (d2) −0.29, Mn-germanene in the energy of (e1) −0.37 and (e2) −0.345, the inset is one dimensional Berry curvature when *k*_*y*_ = 0.

High symmetry point *K* and *K*′ locate on the opposite side of the high symmetric line BD of the reciprocal lattice of 4 × 4 germanene as shown in Fig. 5(a). When we integrate Berry curvature for one valley, the integration can be done only on one side of the line BD, and the integration accuracy of Wannier interpolation have been proved by previous works^41^,^42^. To have a better view of the symmetry to Berry curvature distribution, we used the first Brillouin zone derived from Wigner-Seitz unit-cell in Fig. 6 to plot the Berry curvature. For Ti-germanene, an interesting phenomenon appears from the result of Berry curvature that the sign of its Berry curvature to both valley *K* and *K*′ is reversal if we shift the Fermi level around −0.30 eV, as showed in Fig. 5(b). The reversion of the Berry curvature will produce the reversion of the voltage derived from VHE. However, the PDOS of Fig. 2(a) shows that strong localized *A*_1_ state distributes within the energy window of (−0.45 eV, −0.3 eV). The *A*_1_ state localization destroys the good quantum number of the valleys, namely Berry curvature has finite value contributed by the k-points other than *K* and *K*′, as shown in Fig. 6(a1,a2). Here, we discuss the cause for the emergence of the Berry curvature around Gamma point and its difference from those valley *K*/*K*′. From the inset of Berry curvature line distribution in Fig. 6, we know that the Berry curvature around valley often form extremely sharp peak. But the Berry curvature distribution around Gamma point varies gently in reciprocal space. This phenomenon can be explained by the formula of Berry curvature calculations. According to the formula (1) in the method, Berry curvature can come from two factor: denominator and numerator. If two energy eigenvalues around a k point are very close to each other and the Fermi level lies between them, extremely sharp peak of Berry curvature will form because generally the energy eigenvalues change very fast in reciprocal space around this k point. This is the case for the valley *K* and *K*′ point. If the numerator of velocity operator make big contribution, Berry curvature would be relatively mild, because the wavefunction changes across reciprocal space gently. This is the situation of nonzero Berry curvature around Gamma point for Ti-germannene and V-germanene. Such Berry curvature other than *K* and *K*′ will cover the contribution of the valley *K*/*K*′ to some extent. The system although may show the voltage similar to VHE, the contribution is hardly to be distinguished, which is unsuitable for QVHE. The system V-germanene shares the similar feature with the Ti-germanene as shown in Fig. 6(b1,b2). Because of no global band gap is exist, Ti-germanene and V-germanene are not show any quantization of the Hall conductance. A zoom in band structures around the topological energy window can be found in Fig. S4 in supplementary materials. We should note that, although there are contribution from the k-points other than the valley *K*/*K*′ for Ti-germanene and V-germanene, the main contribution to the Berry curvature still comes from the two valleys. We predict that the Ti-germanene and V-germanene systems are also hoping to exhibit VHE voltage in experiments although it is not purely contributed by the valley polarization.

The Berry curvatures of Cr-germanene when the Fermi energy is −0.39 eV and −0.35 eV respectively are shown in Fig. 6(c1,c2). The AHC with valley-polarized characteristics of Cr-germanene in function of the Fermi level around the Dirac cone are shown in Fig. 5(d). The solid lines in the figure are obtained by fitting scatter data with cubic spline functions. The anomalous Hall conductivities from half Brillouin zone integration almost totally come from the valley *K* and *K*′. AHC contributed by valley *K* keeps positive value in the energy interval we considered. However, the AHC derived from the *K*′ changes from negative to positive within the energy window (−0.36 eV, −0.33 eV). The results indicate that the valley current derived from *K*′ is reversal in the energy window. According to energy band in Fig. 3 and Berry curvature distribution as depicted in Fig. 6(d1,d2), such reversion is closely related to the cross of Fermi level with special energy band at *K*′, which make the Berry curvature around the center zone of *K*′ change its sign. But no essential change is happened for *K* when Fermi level shift from −0.39 eV to −0.35 eV. Interestingly, when the Fermi level is larger than −0.4 eV, the absolute value to the AHC derived from *K* is larger than that of *K*′ producing the total AHC change from negative to positive. When the Fermi level shifts to −0.35 eV, the AHC reaches its maximum. The above results indicate that the AHC can be reversed through shifting the Fermi level, which will produce the reversion of the anomalous Hall voltage (AHV). Such feature of Cr-germanene is very useful for the information devices based on AHE. Similar feature also exhibits in the system Co-germanene. The anomalous Hall conductivities from half Brillouin zone integration almost totally come from the valley *K* and *K*′. AHC contributed by valley *K*′ keeps positive value in the most energy interval we take into account. However, the AHC derived from the *K* changes from negative to positive when the Fermi level is around −0.39 eV. This positive values and positive range are very small, and make the total AHC reach its maximum around the energy. According to the energy band in Fig. 4 and Berry curvature distribution as depicted in the Fig. 6(e1,e2), such reversion is closely related to the cross of Fermi level with special energy band at *K*, which make the Berry curvature around the center zone of *K* changes its sign. But no essential change is happened for *K*′. And then, along with the shift of Fermi level, the AHC derived from *K* increases and reaches its maximum around −0.30 eV. When the Fermi level is larger than −0.30 eV, the total AHC changes from positive to negative. The results indicate that the valley current derived from *K* is reversal similar to Cr-germanene. Actually, when the Fermi level is smaller than −0.48 eV the total AHC of Co-germanene is also negative.

The AHC of Mn-germanene is shown in Fig. 5(f). The values of AHC derived from *K* and *K*′ remain positive and negative, respectively, within the energy window considered in present work. Moreover, the evolution of the AHC derived from K and K′ with the Fermi level shifting to higher energy approximately shows mirror symmetry to zero AHE lines, which results in the total AHC of the system always remains small value in the energy range we considered. We also find that quantum valley Hall Effect occurs for this system, as showed in the inset figure of Fig. 5(f). When the Fermi level is located around −0.35 eV, quantized Hall conductance platform appears for *K* and *K*′ and the value of 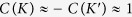. The width of the platform is about 10 meV, which corresponds to the global band gap around the Dirac cone. A zoom in band structures around the topological global band gap can be found in Fig. S4 in supplementary materials.

Now let us summarize the mechanism of diverse behaviors of TM-germanene systems. In pristine germanene, the A and B sub-lattices are equivalent, the valley K and K′ are degenerate and they show same behavior under external perturbation. After TM atom adsorbed on the surface of germanene, this kind of equivalent is broken, and valleys K and K′ show different electronic structure as showed in Fig. 4. The determination of the valley-symmetry breaking can be briefly concluded to two aspects. The first one is the energy distribution of the 3*d* orbitals of TM atom. If the 3*d* orbitals of TM atom is deviate away from the Dirac cone, the Dirac cone and its valley symmetry would largely remain. From the Fig. 4, we find that the energy band of local 3*d* orbitals of Cr-germanene and Co-germanene are away from the Dirac cone of germanene. The electronic states of valley K and K′ of Cr-germanene and Co-germanene only affected by a weak magnetic perturbation. Their valley symmetry is only slightly broken. The second aspect determined the breaking of the valley symmetry is the interaction strength between TM atom and its three NN Ge atoms. Here the bond coupling strength between *A*_1_ of TM and *p*_*z*_ of Ge can be used as the criterion for the extent of broken to valley symmetry. Because the spin-polarized charge mainly comes from the TM atom, and the spin-up state of *A*_1_ are all filled with electron except for Cr-germanene. Moreover, the electronic states of the valleys K and K′ of germanene are mainly derived from the *p*_*z*_ state of Ge. Therefore we can use the degree of deformation of *A*_1_ states to approximately estimate the interaction strength between TM atom and its three NN Ge atoms. Although a local 3*d* orbital passed through the Dirac cone for Mn-germanene, the SCD in Fig. 3(d) tell us that the dumbbell shape *A*_1_ states of Mn is kept very well, which indicates that the interaction between *A*_1_ states of TM and the *p*_*z*_ states of its three NN Ge atoms is weak. The Dirac cone and the valley symmetry of Mn-germanene are kept very well. However, for Fe-germanene, there are not only two local 3*d* states crossed over the Dirac cone, but also great deformation of *A*_1_ states of Fe as shown in Fig. 3(e), indicating strong interaction between *A*_1_ states of TM and the *p*_*z*_ states of its three NN Ge atoms. The Dirac cone and the valley symmetry of Fe-germanenne are nearly completely destroyed. For Ti-germanene and V-germanenne, the interaction strength is fall in between Mn-germanene and Fe-germanene, their Dirac cone and the valley symmetry are partly kept.

Based on the above results, the AHE in TM-germamene can be modulated by shifting Fermi level of the systems. Previous work^43^ has reported that when silicene and BN form superlattice, the electron transfer from silicene to BN leading to the up-shift of Dirac Cone of silicene. We predict that if the TM-germanene grows on a chemical inertia substrate such as BN, the electron transfer between TM-germanene and substrate will shift the Fermi level of TM-germanene and at the same time maintain its property of Dirac cone. Moreover, it has been reported that the Fermi level of two dimensional systems, such as single/bilayer graphene and graphene-*MoS*_2_ heterojunction, can be tuned just by applying gated voltage in the direction perpendicular to the two dimensional plane^44^,^45^,^46^. Therefore, we expect that applying a z-direction gated voltage would be excellent method to tune the Fermi level for our TM-germanene system grown on chemical inertia substrate.

**Figure 7:**
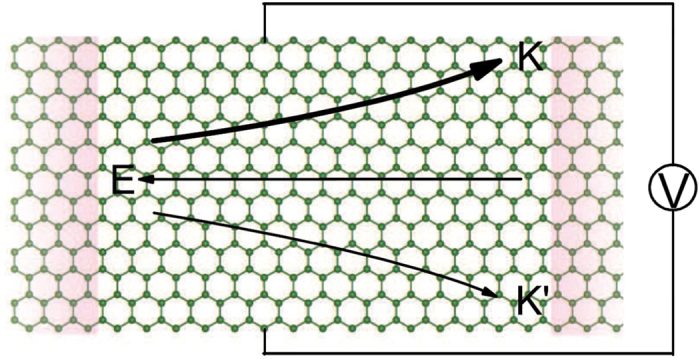
An in-plane electric field will produce a transverse valley voltage between the two edges. Arrow E represents the direction of electric field, arrow *K* and *K*′ express the direction of electronic movement for different valley. The intensity of valley’s AHC is showed by different weighted arrows.

For the common valley Hall materials, such as *MoS*_2_ and graphene, equal amounts of Hall current from each valley flow in opposite directions due to time reversal symmetry, so that no net Hall voltage is produced. In TM-germanene system, this can be easily realized. The mechanism is depicted in Fig. 7. When an in-plane electric field is applied, the electron of different valley acquires opposite anomalous velocity proportional to the Berry curvature in the transverse direction. If the Fermi level shifts to specific energy, unequal Berry curvature between two valleys ultimately leads to a valley Hall voltage between two boundaries. To observe the valley voltage or valley current, we can measure the transverse electronic resistance across the Hall bar, just as previous experimental works^8^,^47^. The measured concrete value can compare with our calculation with the formula 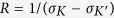. A net valley-polarized electric current can be acquired in the longitudinal direction. Moreover, the AHE of some TM-germanene, such as Cr-germanene and Co-germanene, can be reversed by shifting the Fermi level. Finally, to find out the dependence of the valley polarized Hall effect of TM-germanene on the TM adsorption concentration, we take Cr-germanene with the adsorb concentration of 2.04%, 3.06%, and 3.13% (7 × 7 germanene supercell adsorbed two and three Cr atoms and 4 × 4 germanene supercell adsorbed one Cr atom) as example to calculate their AHC around the Dirac cone. The results can be found in Fig. S5 in the supplementary materials. We find that the concentration will influence the detail band structure of valley, but the valley polarized Hall effect still exist for all the different adsorption concentration.

## Conclusion

On the basis of the first-principles calculations, we report the structure and electronic properties of 3*d* TM adsorbed germanene. Rely on the comparison with previous reports, we find that a proper Hubbard U is important to accurately describe the properties of TM-germanene systems. The Berry curvature calculations indicate that valley-polarized AHE can be realized in the Cr, Mn, or Co adsorbed systems. Furthermore, this kind of valley-polarized AHE can be effectively modulated by shifting the Fermi level of the systems.

## Methods

The electronic structures of germanene adsorbed 3*d* TM atom including Ti, V, Cr, Mn, Fe, and Co were studied with projector augmented wave^48^ (PAW) formalism implemented in the Vienna ab initio simulation package^49^,^50^. General gradient approximation^51^ was used to describe the exchange and correlation energy in the Kohn-Sham equations. The plane-wave cutoff energy was set to be 500 eV and a vacuum space larger than 15 Å was set to avoid the interaction between two adjacent images. The energy convergence criterion was set to 10^−6 ^eV/unit cell. We performed the structure optimization using the conjugated gradient algorithm. All of the atoms were allowed to relax without symmetric restriction until atomic residual forces were smaller than 10^−2 ^eV/Å. To accurately describe the electronic structure around the Dirac point, we considered the semicore state, such as 3*d* of Ge and 3*p* of TM, as valence state in our calculations.

We adopted the GGA + U method in our calculations because the electronic correlation is critical to accurately describe the properties of 3*d* transition metal. Previous work^52^,^53^,^54^ also proved that if proper Hubbard U is added to TM atoms, the value of AHE can be match with the experimental measured value. To determine the parameter U, we used the linear response approach introduced by Cococcioni^33^ implemented in the PWSCF package^55^. In the present work the rotationally invariant DFT + U formalism proposed by Dudarev^56^ was used, where only the value of 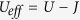 is meaningful instead of individual U and J. To evaluate charge transfer between TM adatom and germanene sheet, we adopted the Bader charge analysis method^57^. By comparing the valence electrons of TM adatom in TM-germanene with its free-standing state, the charge transfer between TM and germanene can be quantitatively determined.

For the part of anomalous Hall conductivity (AHC) calculation, we use the Berry curvature formula:


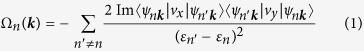



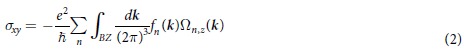


Ω_*n*_ is Berry curvature, *v*_*x*(*y*)_ represents velocity operator, 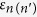 donates the energy of bands calculated in normal first-principles method. As we known there are three main mechanisms for AHE: Intrinsic, Skew-scattering, and Side-jump contribution. We only considered the intrinsic mechanism of the AHE because the topological properties the main topic in present work is mainly determined by the intrinsic one^31^. SOC was used when AHC calculations were performed. To ensure the accuracy of our calculations, we calculate the AHE of Fe and FePt, the results agree well with previous reports^58^,^59^. To obtain accurate AHC, a dense k point mesh is needed which demands amount of calculations with traditional method because it relies on non-self-consistent first-principles calculations^60^. In present work, we used the maximally localized Wannier interpolation^58^ to obtain a high precision Berry curvature distribution in reciprocal space. Moreover, we implemented an adaptive mesh refinement scheme^60^ in k-point space: when the computed Berry curvature of one k point in the original mesh exceeds a threshold value Ω_*cut*_ a refined mesh was applied around this k point, and then the Berry curvature was further integrated under this refined mesh. In present work, the k-point mesh of 5 × 5 × 1 was used in VASP calculations, and then we interpolated the k point mesh to 60 × 60 × 1 in reciprocal space to obtain the Berry curvature. If the value of Berry curvature of one k point exceeds Ω_*cut*_ = 90 a.u., a refined submesh of 5 × 5 × 1 around the k point was used. The parameters used in our calculation were tested (100 × 100 × 1 and 200 × 200 × 1 interpolated mesh were used) and they are enough to achieve the precision of 0.1 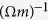 for AHC.

To evaluate the relative stability of different adsorption sites, we calculate the adsorption energy of TM-germanene system defined as:


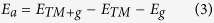


The terms *E*_*g*_, *E*_*TM*_, and *E*_*TM*+*g*_ represent the total energies of the bare germanene, the free TM atom, and the TM-germanene system, respectively. The smaller *E*_*a*_ means more stable structure. In present paper, 4 × 4 supercells of germanene as shown in Fig. 1(a) was employed to avoid TM atom interaction with it periodic images.

## Additional Information

**How to cite this article**: Zhou, P. and Sun, L. Z. 3*d* Transition Metal Adsorption Induced the valley-polarized Anomalous Hall Effect in Germanene. *Sci. Rep.* **6**, 27830; doi: 10.1038/srep27830 (2016).

## Supplementary Material

Supplementary Information

## Figures and Tables

**Table 1:** Results for single 3*d* TM atom adsorbed germanene.

**TM**	***U***(**eV**)	***E***_***b***_(**eV**)	***E***_***b***_(***U***) (**eV**)	**d**(***TM*****–*****Ge***_***A***_) (**Å**)	**d**(***TM*****–*****Ge***_***B***_) (**Å**)	**M**(***μ***_***B***_)	***T***_***e***_
Ti	4.666	4.807	2.579	2.323	2.571	2.67 (1.13)^37^	−1.153
V	3.868	4.274	3.478	2.530	3.073	4.67 (3.01)^37^	−0.790
Cr	6.360	1.489	1.996	2.508	3.036	2.84 (4.00)^37^	−0.672
Mn	4.732	3.821	1.745	2.414	2.844	5.75 (4.95)^37^	−0.626
Fe	6.319	3.306	3.205	2.408	2.831	3.04 (3.24)^37^	−0.567
Co	5.924	4.146	2.492	2.321	3.530	2.03 (1.10)^37^	−0.285

The results contain the Hubbard U of 3*d* orbits of TM (U), adsorption energy of 3*d* TM without Hubbard U(*E*_*b*_) and with Hubbard U(*E*_*b*_(*U*)), distance between the adatom to its nearest Ge atoms of sublattice A/B (dTM-*Ge*_*A*_/*Ge*_*B*_), magnetic moment per unit cell (M), and charge transfer from the TM to the germanene (*T*_*e*_).
