# Supplementary material for: 3d Transition Metal Adsorption Induced the valley-polarized Anomalous Hall Effect in Germanene
Source: Sci Rep. 2016 Jun 17;6:27830. doi: 10.1038/srep27830 (PMC4911552; doi:10.1038/srep27830)
Supplement: Supplementary Information [file srep27830-s1.pdf]

$3d$ Transition Metal Adsorption Induced Vally-polarized Anomalous Hall Effect in Germanene

P. Zhou[1] and L. Z. Sun[1,*]
[1]*Hunan Provincial Key laboratory of Thin Film Materials and Devices, School of Material Sciences and Engineering, Xiangtan University, Xiangtan 411105, China*
(Dated: May 18, 2016)

PACS numbers: 71.20.-b, 71.70.Ej, 73.20.At

To analysis the cluster formation on the surface of germanene, we take the Cr-germanene system as typical example and take into account four top position, they are indicated with black letters in Fig.S 1. We label them topA, topB, topC, and the top position of the first TM atom topT. After structure relaxation, we found the TM atom in topC position relax to topB position. So there are only three positions at last. We calculated the adsorption energy of all TM dimer configurations with GGA+U method because the adsorption configuration is sensitive to the Hubbard U. By comparing the energy of these three positions, we find the order of energy follow $E_{topB} < E_{topC} < E_{topA}$. Because of the energy of topA is nearly 2.0 eV higher than that of topT, we mainly consider the topB and topT positions. With the stable structure of the second TM atom located on topB and topT, we did first-principles molecular dynamics calculation to these two cases with the temperature of 500K. The results show that, if the second TM atom added on the top of first TM atom, the first TM atom would pass through the Ge six-ring and form the sandwich structure. We calculated its energy band with SOC and its AHC around Dirac cone and found that its electronic structure around K and K share the same characteristics. The intrinsic AHC around Dirac cone almost disappears. According to the topB configuration, it is 1.05 eV more stable than the topC, indicating the most stable TM dimer structure. Therefore, the dimer of TM can be formed on the surface of germanene. In order to analysis the impact of TM dimer on the AHE of TM-germanene system, we also calculated its energy band with SOC and AHC around Dirac cone. The results show that, compared with the single Cr-germanene, the second Cr adjust the Fermi level to the energy range of pristine germanenes Dirac cone. Although the second Cr atom introduces localized states of 3d orbitals near Dirac Cone, the Berry curvature extremes distribute around the valley (K and K), we expect the dimer would not damage the valley-polarized AHE. However, the concrete AHE would change with the formation of TM dimer. All in all, although the TM dimer can form on the surface of Cr-germanene, the valley-polarized AHE modulation to germanene still exists.

In the present work, we used the linear response theory introduced by Cococcioni et al[1], which has been successfully applied in TM atom- and dimer-doped two dimensional systems[2,3]. The U parameter can be calculated directly by a response function as follows:

$$\chi_{ij} = \frac{dn^i}{d\alpha_j}, \chi^0_{ij} = \frac{dn^i_0}{d\alpha_j} \tag{1}$$

In the linear-response approach, χ_{ij} and χ^0_{ij} are obtained from the response of d state occupations to a small localized perturbation potential α_j , and then the parameter U can be obtained from the following formula:

$$U = \chi_0^{-1} - \chi^{-1} \tag{2}$$

By changing the rigid potential α_j, we obtain the bare and self-consistent occupation regression response functions. The interacting (χ_{ij}) and the Kohn-Sham (χ^0_{ij}) inverse matrices are the slopes of the bare and self-consistent regression response functions, respectively, as shown in Fig.S 2. All of them are obtained with the self-consistent calculations in first-principle method. And then with the help of formula (2), we can obtain the Hubbard U of 3d TM atoms.

The zoom in energy band with SOC for Mn-germanene and V-germanene are depicted in Fig.S 4. They clearly show that an approximate 10 meV global gap is exist for Mn-germanene, quantum platform is possible for it. However, no global gap is happened for V-germanene, therefore no quantum phenomenon appears for V-germanene.

To simulate the different adsorption concentration of TM atoms, we take the Cr-germanene as an example to illustrate the concentration effect. Fig.S 5 shows the results of 2.04%, 3.06% and 3.13% adsorption concentration. We find the Dirac cone locate different energy interval with different concentration. We also find that the concentration have different influence to different valley, but the valley polarized Hall Effect still exist all concentrations.

---

* Electronic address: lzsun@xtu.edu.cn

1 Cococcioni, M. & de Gironcoli, S. Linear response ap-

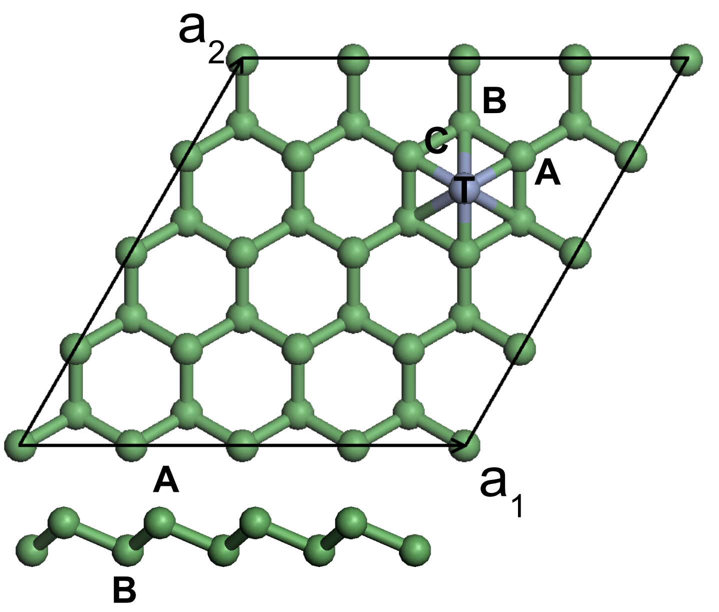

Fig.S 1: (Color online)Top view of germanene monolayer where 4 adsorption sites topA, topB, topC and topT are marked out with black letters. The lower panel of (a) is the side view of germanene

proach to the calculation of the effective interaction parameters in the LDA+U method. *Phys. Rev. B*, **71**, 035105; DOI:10.1103/PhysRevB.71.035105 (2005).

[2] Wu, M., Cao, C. & Jiang, J. Electronic structure of substitutionally Mn-doped graphene. *New J. Phys.*, **12**, 063020; DOI:10.1088/1367-2630/12/6/063020 (2010).

[3] He, J. et al. Magnetic Exchange Coupling and Anisotropy of 3d Transition Metal Nanowires on Graphyne. *Sci. Rep.* **4**, 4014; DOI:10.1038/srep04014 (2014).

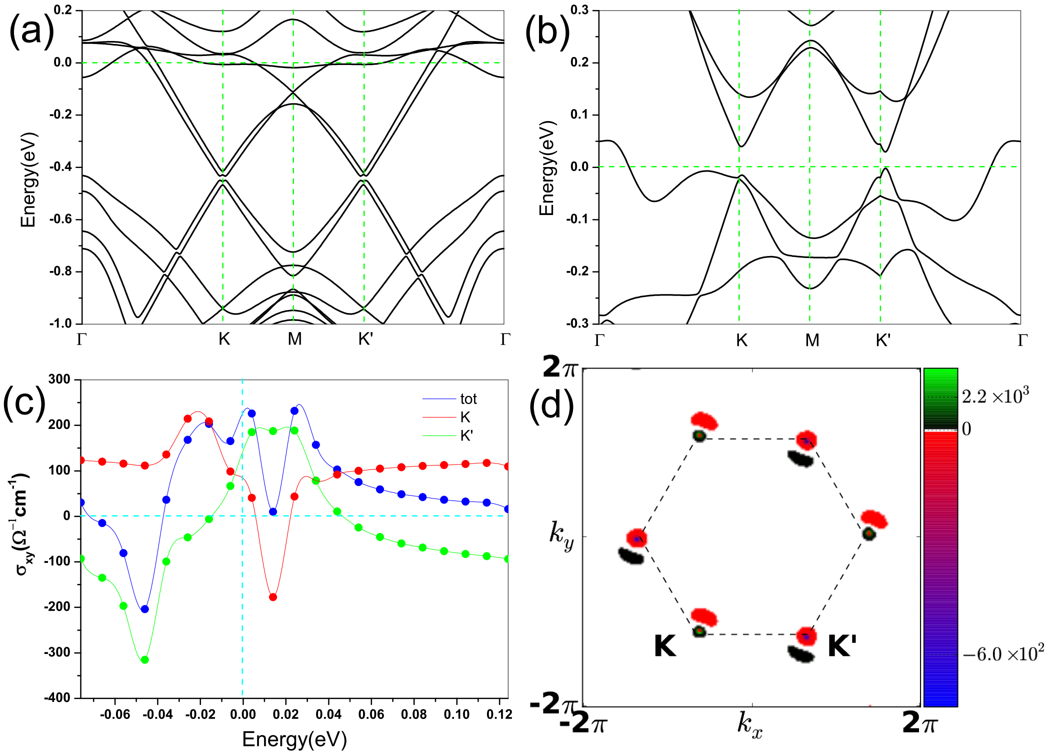

Fig.S 2: (Color online)Energy band with SOC for (a) 2Cr-germanene-T4 and (b) 2Cr-germanene-T1. (c) AHC around the Dirac cone for 2Cr-germanene-T1. (d) The distribution of the Berry curvature in momentum space for 2Cr-germanene-T1 in the energy of Fermi level

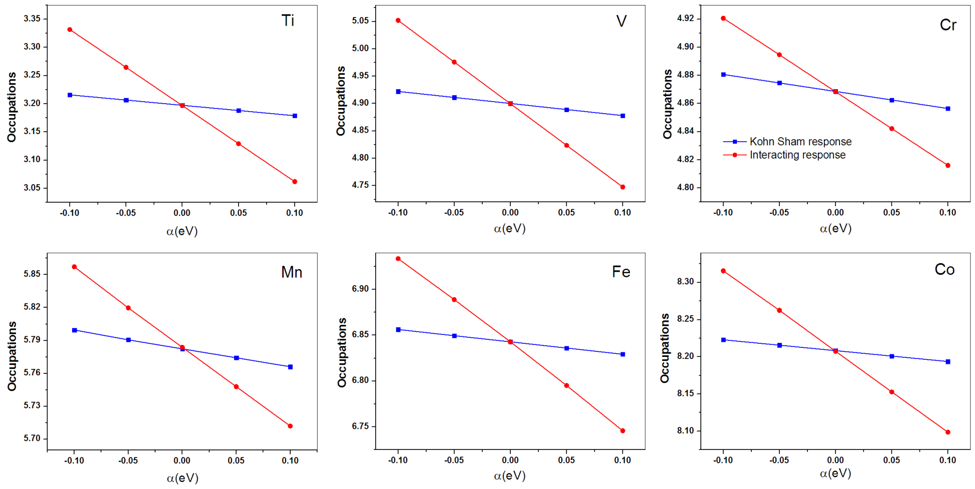

Fig.S 3: (Color online)The change of occupation with perturbation of α. The TM atom are label on the top-right side of graph.

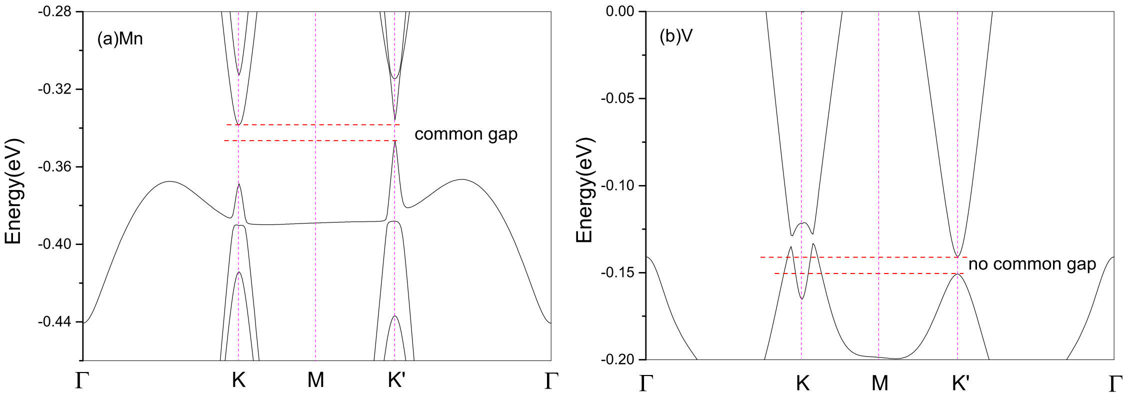

Fig.S 4: (Color online)Zoom in energy band with SOC for (a) Mn-germanene and (b) V-germanene.

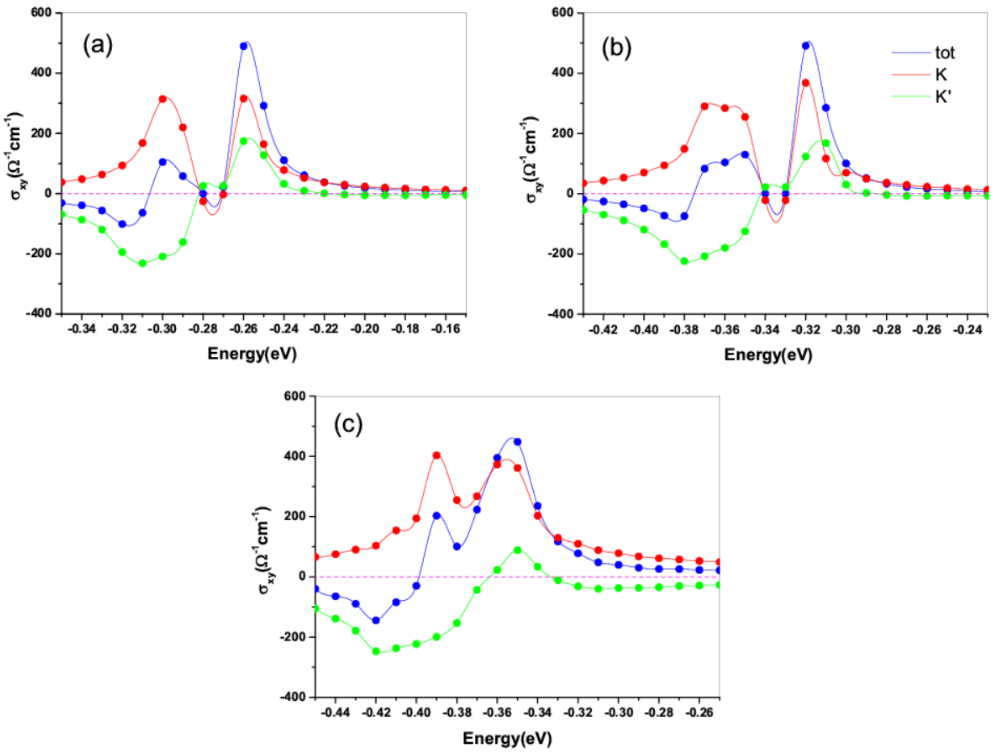

Fig.S 5: (Color online)AHC around the Dirac cone for Cr-germanene with the adsorb concentration of (a) 2.04% (b) 3.06% and (c) 3.13%. The Fermi energy is set to zero.

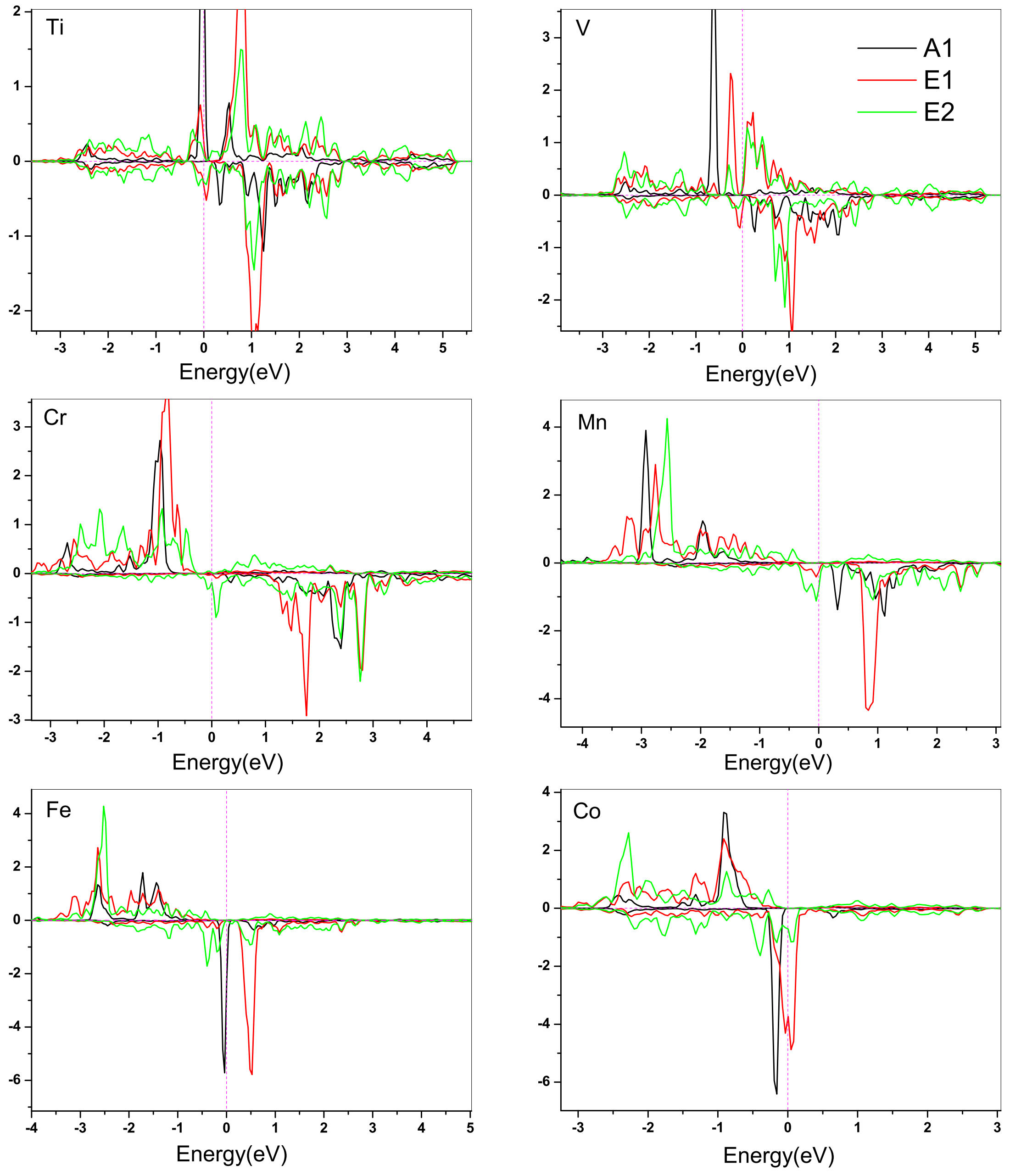

Fig.S 6: (Color online)Projected density of states for TM-germanene system without Hubbard U. The positive and negative values denote spin-up and spin-down channels, respectively. The Fermi energy is set to zero.
